# LRH-1 induces hepatoprotective nonessential amino acids in response to acute liver injury

**DOI:** 10.1172/JCI168805

**Published:** 2023-04-03

**Authors:** Kevin C. Klatt, Elizabeth J. Petviashvili, David D. Moore

**Affiliations:** Department of Nutritional Sciences & Toxicology, University of California Berkeley, UCB, Berkeley, California, USA.

## Abstract

Acute hepatic injury is observed in response to various stressors, including trauma, ingestion of hepatic toxins, and hepatitis. Investigations to date have focused on extrinsic and intrinsic signals required for hepatocytes to proliferate and regenerate the liver in response to injury, though there is a more limited understanding of induced stress responses promoting hepatocyte survival upon acute injury. In this issue of the *JCI*, Sun and colleagues detail a mechanism by which local activation of the nuclear receptor liver receptor homolog-1 (LRH-1; NR5A2) directly induces de novo asparagine synthesis and expression of asparagine synthetase (ASNS) in response to injury and show that this response restrains hepatic damage. This work opens up several avenues for inquiry, including the potential for asparagine supplementation to ameliorate acute hepatic injury.

## Hepatic injury and regeneration meet asparagine

It has been nearly 100 years since the pioneering work of Higgins and Anderson demonstrated the liver’s remarkable capacity for regeneration following partial hepatectomy (1), launching intense investigation into the cell-autonomous and noncell-autonomous factors required to respond to injury and return to homeostasis. Liver injury is commonly modeled through surgery (e.g., partial hepatectomy [PH] or bile duct ligation) and chemically, via administration of acute or chronic hepatotoxic compounds (e.g., dimethylnitrosamine, carbon tetrachloride, and acetaminophen) (2–4). Such models of liver injury exhibit an early initiation phase characterized by cytokine response, which is concordant with the entry of quiescent (G0) hepatocytes into the cell cycle and their subsequent progression through it. Chemically induced injury models, specifically, begin with a period of hepatocyte apoptosis and tissue damage prior to hepatocyte proliferation, thus allowing for a more translational model of acute hepatic injury. Models of liver injury have made great strides in identifying signals, such as growth factors, cyto- and chemokines, and hormones, required for normal hepatocyte proliferation and hepatic regeneration, and defining complete mitogens required for regeneration and numerous auxiliary mitogens that modify the time course of regeneration (5). In contrast, only a limited body of literature has investigated factors that prevent hepatocyte cell death prior to hepatocyte proliferation, providing limited insight into physiologically relevant stress responses and potential therapeutic avenues for acute liver injury management (6–9). In this issue of the *JCI*, Sun, et al. advance our understanding of such prosurvival signals in response to hepatocyte injury, delineating a mechanism by which hepatocyte activation of the nuclear receptor liver receptor homolog-1 (LRH-1) rewires metabolic networks to drive asparagine synthesis from glutamine, a process that limits the extent of hepatocellular death and liver injury without affecting proliferation (10).

Sun and colleagues dissected asparagine’s hepatoprotective roles following the curious observation that the highly zonated processes of glutamine metabolism in the liver were further compartmentalized at the cellular level. Hepatocytes exhibiting the transcriptional machinery necessary to synthesize glutamine (termed GLUL-enzyme positive, or GLUL^+^) also expressed asparagine synthetase (ASNS), but lacked glutaminase, glutamine’s primary catabolic enzyme in the cell. Sun et al. demonstrated that ASNS, the enzyme required to synthesize asparagine from glutamine and aspartate, was maximally induced upon sublethal dosing of carbon tetrachloride (CCl_4_) or acetaminophen 24 hours after administration. The observation that genetic ablation of hepatic ASNS enhanced liver damage in both models confirmed that this response was adaptive. Intriguingly, ASNS was not induced through classical cellular stress response pathways (e.g., ATF4) but rather through the activity of LRH-1. Hepatic LRH-1–knockout mice exhibited low ASNS expression and increased damage in response to chemically induced hepatic injury. Conversely, two mouse models of LRH-1 gain-of-function — the activating LRH-1 K289R point mutation and knockout of hepatic small heterodimer protein (SHP), a negative regulator of LRH-1 — exhibited upregulated ASNS expression and showed substantial protection from hepatic injury. ChIP and cotransfection assays confirmed that LRH-1 bound directly to the ASNS promoter and increased transcriptional activity. Since genetic ablation of hepatic ASNS alone could not indicate the relative impact of either depletion of substrate or generation of product, Sun et al. confirmed, via amino acid profiling and evaluating the consequences of i.v. injection, that the ASNS product asparagine, but not glutamate or valine, was indeed the protective metabolite (10).

Sun and colleagues focused their experimental work on acute hepatotoxic injury models in mice, but also raised the prospect of broader translation by identifying increased ASNS expression in human cohorts of pharmaceutical, environmental, and viral hepatic injury that suggest induction of asparagine as a potential common hepatoprotective response to hepatic stressors (10). Collectively, these results build on several emerging areas of metabolic biology worth highlighting.

## Metabolic signaling

After decades of being thought of as passive substrates in cellular anabolism, catabolism, and whole-body nitrogen and carbon handling, amino acids are emerging as potent regulators of cell function and fate that require fine-tuned sensing machinery. The now-classic example is leucine, sensed by the serine-threonine-protein kinase GCN2 and signaling through mTORC1 via alleviation of sestrin-mediated inhibition. Further roles for essential and nonessential amino acid signaling and determination of cell fate continue to emerge, particularly in the field of cancer metabolism. Prior to the investigation by Sun et al., little was known about cellular signaling roles of the nonessential amino acid asparagine, apart from early observations that asparagine inhibits autophagic protein breakdown via lysosomal delivery (11). The results by Sun et al. raise provocative questions about how asparagine exerts its hepatoprotective effects (10). Asparagine’s effects may be direct, through autophagy-related or currently unrecognized prosurvival effects, or they may be indirect. Indeed, in the field of cancer metabolism, asparagine has been recognized as a proproliferative factor secondary to its role as an amino acid exchange factor, whereby intracellular asparagine export results in serine/threonine uptake, mTORC1 activation, and coordinated protein and nucleotide synthesis (12). The results by Sun, et al. (10) warrant a renewed interest in asparagine and the mechanisms by which it induces a prosurvival hepatocyte program.

## Revisiting nuclear receptors in hepatic injury and regeneration

The role of LRH-1 in promoting ASNS expression in the hepatocyte builds on existing research highlighting the importance of nuclear receptors in coordinating responses to liver injury. For example, both hepatic farnesoid X receptor (FXR) and the constitutive androstane receptor (CAR) expression have been shown to affect liver regeneration following injury, linking both metabolic signals — i.e., bile acids — and xenobiotic metabolism to injury and repair (13, 14). PPAR α-induced autophagy has also been shown to be protective in acute liver failure models (15, 16). LRH-1 has received little investigation in the context of nonmetabolic disease-related liver stresses, with a single investigation revealing that hepatic LRH-1 knockout mice exhibit increased ER stress and liver fat accumulation 48 hours after PH (17). The findings in Sun, et al. suggest that LRH-1 has a unique role in promoting an early hepatocyte prosurvival response — rather than hepatocyte proliferation, per se — highlighting a time-course dependency of different nuclear receptors in the hepatic injury and regeneration cycle. While some work has previously indicated LRH-1 regulates one-carbon metabolism-related amino acids (18, 19), the data from Sun et al. raise important questions about LRH-1’s role in sensing cellular stress, as well as the upstream factors required to mobilize LRH-1 to the ASNS locus. The role of LRH-1 in responding to hepatic injury readily brings to mind its role in the enterocyte, where LRH-1 has well-accepted functions in responding to inflammatory stimuli, inducing an antiinflammatory program to resolve inflammation and limit cell damage (20, 21). How injury and other homeostatic stressors, such as feeding (22), induce LRH-1 remains an open question; LRH-1 activity is regulated by ligand-binding, posttranslational modifications, and protein-protein interactions (22, 23), all or some of which may exhibit relevant changes in response to stress signals. Future work is needed to both define these relevant signals and determine whether LRH-1 ligands may play a therapeutic role in acute hepatic injury (24, 25).

## Nutritional and metabolic support during injury

Unlike ASNS’s product asparagine, its substrate glutamine has been intensively studied in both cellular and clinical contexts. Interest in glutamine stems from early observations by Bergstrom and colleagues (26) that free glutamine is reduced in skeletal muscle following surgery. The work spawned decades of investigation into intra- and inter-organ amino acid fluxes during injury and illness (27, 28) and research on the uniquely protective roles of glutamine in various processes, including immune cell proliferation and function, cell swelling-related inhibition of proteolysis, activation of anabolic processes, expression of the stress sensor Hsp70, and synthesis of glutathione (via glutamate) (29). The work by Sun and colleagues suggests that glutamine’s metabolic fate of asparagine synthesis may provide an additional mechanism by which glutamine administration, outside the context of hyperammonemia, could be considered hepatoprotective. Whether glutamine can substitute for asparagine in inducing this hepatoprotective program is critical to explore further, as asparagine is absent from most parenteral amino acid solutions, and stable dipeptide formulations of glutamine are readily available. Regardless, the results of Sun, et al. highlight the need for the clinical nutrition community to consider the therapeutic potential and possible conditional essentiality of asparagine in the context of various acute hepatic stressors. As coexpression of ASNS and LRH-1 exist in other tissues at risk of injury — e.g., pancreas — further investigation is needed to determine whether asparagine has broader relevance beyond the hepatocyte as well.

## Conclusion

Collectively, the results by Sun and colleagues identify an endogenous hepatoprotective program relying on de novo asparagine synthesis induced by LRH-1 in the hepatocyte to promote cell survival in response to injury (10). These results prompt several questions about the mechanisms by which asparagine promotes cell survival; whether hepatocytes sense cellular asparagine; the signals that induce LRH-1 activity upon injury; and the potential clinical implications of providing this nonessential amino acid.

## Version 1 04/03/2023

Electronic publication
